# Big data-driven intelligent governance of college students' physical health: System and strategy

**DOI:** 10.3389/fpubh.2022.924025

**Published:** 2022-08-09

**Authors:** Chenliang Deng, Qiaoyan Yu, Ganglin Luo, Zhangzhi Zhao, Yuchao Li

**Affiliations:** ^1^Sports Department, University of Electronic Science and Technology of China, Chengdu, China; ^2^School of Gymnastics, Chengdu Sport University, Chengdu, China; ^3^Department of Military Education and Training, Police Academy of the Armed Police, Chengdu, China

**Keywords:** information technology, big data, information physical education, physical health of college students, intelligent governance of physical health

## Abstract

With the development of information technology, the application of a new generation of information technologies, such as big data, Internet Plus, and artificial intelligence, in the sports field is an emerging, novel trend. This paper examined the relevant research results and literature on physical education, computer science, pedagogy, management, and other disciplines, then used a self-made questionnaire to investigate the physical health status of Chinese college students. The big data were subsequently analyzed, which provided a scientific basis for the construction of an intelligent governance system for college students' physical health. Intelligent devices may be used to obtain big data resources, master the physical sports development and psychological status of college students, and push personalized sports prescriptions to solve the problems existing in college students' physical health. Research shows that there are four reasons for the continuous decline in Chinese college students' physical health levels. These are students' lack of positive exercise consciousness and healthy sports values (85.43%), a weak family sports concept and lack of physical exercise habits (62.76%), poor implementation of school sports policies (55.35%), and people's distorted sports value orientation (42.27%). Through the connecting effect of data, we can bring together the positive role of the government, school, society, family, and students so as to create an interlinked impact to promote students' physical health. The problems of insufficient platform utilization, lack of teaching resources, lagging research, and insufficient combination with big data in the intelligent governance of physical health of Chinese college students can be solved by building an intelligent governance system of physical health. Such a system would be composed of school infrastructure, data resources and technology processing, and intelligent service applications. Among these, school infrastructure refers to the material foundation and technical support. The material foundation includes perceptions, storage, computing, networks, and other equipment, and the technical support includes cloud computing, mobile Internet, the Internet of Things, artificial intelligence, and deep learning. Data resources refer to smart data, such as stadium data, physical health management data, and students' sports behavior data, which are mined from data resources such as students' physical development, physical health, and sports through big data technology and intelligent wearable devices. Intelligent managers provide efficient, intelligent, accurate, and personalized intelligent sports services for college students through data resource value mining, venue space-time optimization, health knowledge discovery, sports prescription pushes, etc. Finally, we put forward the development strategy for further deepening and improving the big data-driven intelligent governance system for college students' physical health. The intelligent governance system of physical health driven by big data and its development strategy can not only accurately guide and improve the physical health level of college students but also realize integrated teaching inside and outside physical education classes.

## Introduction

Physical health is the basic right and the first wealth of college students and, more importantly, the basic premise of their healthy study, work, and life in the future. Therefore, how to improve the physical health level of college students is a very meaningful and valuable work pursued by the country, school, society, family, and students.

During the past 70 years, since the founding of the People's Republic of China, the party and the state have remained concerned and attached great importance to the health of students. To encourage and promote students to actively participate in physical exercise and enhance students' physical fitness, the relevant competent departments developed a series of systems in different periods, such as the “Labor Hygiene System” (1954), “National Physical Exercise Standards” (1975), “Student Physical Education Qualification Standard” (1990–1991), and “Sports Examination Methods for Junior High School Graduates” (1997). In 2002, the “Standards for Students' Physical Health” was piloted in schools across the country and has been implemented for more than 10 years. In 2014, the Ministry of Education issued a new “Standards for Students' Physical Health,” which required that “all schools carry out physical health tests covering students of all grades every school year and stipulates that students whose physical health test score is <50 points will be treated as lacking completion or having incomplete study and unqualified schools will be rejected in the evaluation.” In 2016, the Opinions on Strengthening School Physical Education and Promoting the All-round Development of Students' Physical and Mental Health contended that “we should carry out strategic, forward-looking, and applied project research aimed at improving students' physical health, teaching quality, after-school training, and sports cultural level through multiple channels so as to improve the overall level of school physical education work.” In 2018, President Xi Jinping stressed at the National Education Conference that “health comes first in education, and physical education classes should be provided to help students enjoy fun, enhance their physical fitness, improve their personality, and temper their willpower.” In 2020, the Opinions on Comprehensively Strengthening and Improving School Physical Education in the New Era clearly pointed out that “under the guidance of Xi Jinping Thought on Socialism with Chinese Characteristics for a New Era, by fully implementing the party's education policy, adhering to the socialist direction of school running, and taking moral education as the fundamental and socialist core values as the guidance, we aim to serve the all-round development of students and enhance their comprehensive quality, adhere to the education concept of health first, and promote the coordinated development of cultural learning and physical exercise for young people.” In the same year, the “Notice on Deepening the Integration of Sports and Education to Promote the Healthy Development of Teenagers,” which was jointly issued by the General Administration of Sport of China and the Ministry of Education, also suggested to “establish the education concept of health first, then open full physical education for all students, to help students enjoy fun in physical exercise, enhance their physical fitness, improve their personality, temper their will, realize the civilization of their spirit, and savage their body.” In 2021, the “Opinions on Comprehensively Strengthening and Improving School Health and Health Education in the New Era” suggested to “implement the fundamental task of moral education, adhere to the education concept of health first, and comprehensively improve students' health literacy into a high-quality education system as an important goal and evaluation standard of school education. We will deepen the reform of health education in schools; promote students' physical and mental health, develop a healthy lifestyle; and train them to be socialist builders and successors who are well-developed morally, intellectually, physically, aesthetically, and laboriously.” The implementation of a series of national policy documents and systems has pointed out the direction for the development of physical education in schools, and it is of great significance to further strengthen the physical education of young students, enhance their physique, and vigorously promote quality-oriented education.

At present, domestic experts and scholars' research on big data sports is still in the initial stage of exploration, and some research results have been accumulated. The research directly related to this paper mainly includes four aspects, one of which is research on big data in sports. Since 2013, big data in sports has become a hot topic widely discussed. In 2015, the five trillion sports industry's goal was to attract Internet giants to break into big data of physical layout, but the research on theory and practice is limited and only a small number of researchers' findings have focused on big data in sports, new ecosystems, forward-looking analysis, sports communication, etc. The second aspect is the research on the sports industry against the background of big data. Representative research in this field includes work by Tong et al. (1), Dong et al. (2), Yu (3), Xie et al. (4), and other documents and materials. This research has mainly focused on the development of the sports industry in the era of big data, including opportunities and challenges, paths, and financial risk prevention and control as well as facility operation, operation for sporting events, sports marketing, sports industry integration, and so on. The third aspect is the research on physical education in the era of big data. Currently, there are only a few studies, mainly those of Zhang and Sun (5). The research results mainly discuss the application of big data in school physical education, the reform of physical education teaching methods, the change of physical education learning methods, and the precision teaching mode of physical education from the aspects of appeals, trends, and action. Fourth is the study of intelligent sports. Representative research results in this aspect are concentrated in the areas of intelligent sports classrooms (6), intelligent sports stadiums (7), intelligent sports services (8), and intelligent governance of public sports services (9). Experts and scholars have conducted their research from the perspectives of the Internet, big data, the mobile Internet, cloud platforms, and artificial intelligence. Overall, the related research results for this research laid the foundation and provide a theoretical reference. However, available big data technology research to improve the physical health of university students is limited; the qualitative-quantitative combination of sports science and medicine is lacking; the combination of computer science, management, sociology, and related influencing factors of college students' physical health in comprehensive research is unexplored; and there is no research on college students' physical health service system driven by big data.

With the in-depth development of information technology, big data has brought new development opportunities to all walks of life, and school physical education is also currently facing many opportunities and challenges. The integrated development of school physical education and information technology has become a core task and urgent demand to solve the existing problems of school physical education in China. At present, the development of school sports informatization in China is poor, lacking both talents and technology, which does not match the current national strategic environment. Given the current research and development situation, how to integrate college students' physical health and college sports resources with computer technology, physical health services, and other elements remains challenging. Equally daunting is how to build a big data-driven college students' physical health service system for management coordination, decision support, intelligent governance, and the construction and development of college students' intelligent sports venues. Therefore, based on the challenges of college students' physical health under the current big data environment and the relevant research status of intelligent governance, this study starts from the key integration of advanced information technology, such as big data, and the development of student's physical health. It carries out innovative research in theory, technology, application, evaluation, and other aspects, and improves the level of college students' physical health through data accuracy technology to build an intelligent governance system for college students' physical health based on big data.

## Methods

Based on the theoretical and practical knowledge of school physical education, computer science, pedagogy, management, and other disciplines, this paper studied the research results and available literature on college students' physical health at home and abroad and used existing theoretical and practical results to support and build the theoretical framework and methodology of this paper.

By using self-compiled questionnaires to investigate the physical condition of college students and through big data analysis and sorting, this study provides a scientific basis for building an accurate system for the improvement of college students' physical health. To collect and assess students' physique, health, and influencing factors, the head of the department in charge of local sports and sports science, management, and computer science or a scholar of related fields visited the relevant domestic school to conduct a full investigation.

The influencing factors of this study are determined by a factor analysis method. Based on the contents obtained from the literature discussion and interviews with experts and scholars, questionnaires were designed according to the preliminarily constructed influencing factors of college students' physical health; three rounds of expert surveys were undertaken to establish the influencing factors. In the study of students' physique health service systems, determination by wisdom is one of the most widely used methods in social science research employing the Delphi method. Through literature review, interviews of experts, and based on the content according to the preliminary build of the students' physique health service system, designed the questionnaire, performed three rounds of expert investigation, and established the system.

We grasped students' physical sports development, and psychological conditions by using emerging information technology that pushed personalized teaching and sports prescriptions, and addressed the existing problems of college students' physical health by using big data technology. We summarized and studied while providing timely feedback and revision of the plan, and performed the experiment laid out in this study.

## Results

### Analysis of reasons for the decline in Chinese college students' physical health

In recent years, the physical health level of Chinese college students has continued to decrease due to the effect of subjective and objective factors of the government, schools, society, families, and students.

#### Subjective factors of declining physical health of college students

According to the survey results, at present, 85.43% of students in Chinese universities lack active sports consciousness and correct values. In other words, they do not know why they want to exercise. As Mao Zedong said in the vernacular translation of the Study of Sport: to deal with a matter is reflected in behavior; we must first start to be interested in or like it. In particular, we must first have a specific understanding of the matter and why such wisdom is necessary and must clearly understand the specifics and why, which is the embodiment of self-consciousness. Many college students do not know the benefits of sports for themselves, either very well or not at all. In short, the stimulation of sports potential is inadequate, so there is no emotion to mobilize sports interest. Those students who can study tirelessly and assiduously think that the relationship between sports and learning is not close, and their survival is not based on sports. Some even think that sports will delay their cultural learning and other activities. Therefore, sports self-consciousness is resulting in worrying physical health. This portion of the reason for non-participation should be blamed on students' inability to deeply understand the benefits of sports. However, teachers do not know how to enlighten students or how to work hard ideologically, which also accounts for half of the responsibility. On the other hand, students lack correct sports values. They are embarrassed to exercise and consider people who can exercise stupid. This is the big reason why people do not like sports. Gentle and slow people look very good and are also respected by society. What does a person who suddenly opens their arms and feet, stretches their limbs, and bends their body look like? Shouldn't this be considered particularly strange? So, there are people who know that the body must move and want to take action but don't take action. There are also people who can exercise together but can't exercise alone, and then there are those who can exercise in their own home but can't exercise in places with many people (10). In short, the subjective reason for the decline of college students' physical health is that they are self-conscious and shy, do not form the habit of physical exercise, and do not establish a correct concept of physical education.

#### Objective factors of declining physical health of college students

One objective factor at play is the concept of family sports and exercise habits, which accounts for 62.76%. Family is the main place where students grow up and live and also the important base of students' enlightenment and education (11). In a sense, the concept of family sports, sports atmosphere, and sports habits will directly affect the enthusiasm of students in participating in physical exercise. Specifically, if the family has good sports traditions and a physical exercise atmosphere and parents generally like to participate in sports fitness activities, students will naturally fall in love with sports fitness activities in college. These individuals tend to experience the passion and fun of sports in a family atmosphere. On the other hand, if the family does not have the atmosphere and tradition of sports activities, their participation in sports activities will inevitably be less. In effect, the concept of “preconception gives priority to” decides the outcome. Compared to school education, family education is more likely to stimulate students' enthusiasm and participation. Family intimacy, tacit understanding, and other factors determine the important position of family education in cultivating students' good physical exercise habits (12). According to a 2019 survey report, 64.8% of Chinese parents said physical health is always the top priority, while 75.9% of parents of kindergarteners and >50% of parents in other learning stages support physical education. In addition, 73.6% of parents believe a lack of exercise is the main reason for college students' poor physical fitness. However, although the importance of physical development is recognized, in the actual home environment, the lack of momentum is very serious. A family's poor sports atmosphere and lax education are common phenomena. On the one hand, parents exercise an average of 2.5 h a week, and >60% exercise with their children no more than once a week. On the other hand, 66.8% of parents have adopted a relaxed educational approach to cultivate their children's sports habits, focusing on their children's interests rather than discipline. Only 1/3 of parents adopt a strict physical exercise regimen. The decrease in exercise time and frequency is directly reflected in the physical condition of students, resulting in the continuous decline of students' physical health levels. Parents rate their children's physical health as passing, not good. Parents of high school students awarded the lowest scores for their children's physical health.

Second, the school sports policy implementation deviation accounts for 55.35%. According to the survey results, most colleges and universities have problems with the implementation of school sports policies, be it in its scale, insufficient depth, insufficient strength, or false implementation methods. In fact, most schools have not really implemented relevant sports policies, which has led to a sharp decline in the physique of Chinese teenagers to a certain extent. Additionally, the school does not pay enough attention to the cultivation of core literacy regarding physical fitness. The study of cultural courses occupies a large portion of the college students' spare time and increases with the advancement in grade, resulting in the lack of exercise time among college students. Nearly 67.6% of college students exercise no more than twice a week. The above problems are caused by the issues in school sports policies, their weak implementation, and the lack of an effective supervisory mechanism. Therefore, we should improve and optimize the level of policy formulation, enhance capacity for policy implementation, identify and support policy implementation objectives, provide resources and the environment for policy implementation, and have a monitoring and oversight system for policy implementation (13).

Finally, the influence of distorted sports value orientation of social groups accounts for 42.27%. In China, although people know they should be active in sports and believe that promoting sports can make a country strong, they tend to pay more attention to cultural education than physical education. Such ideas and actions lead most people to hover between welcoming and rejecting sports. So, most people do not really like sports. Second, most of today's educators are not good at sports. They don't know much about sports and may have only heard of the word “sports,” so when they come to teach sports, they start from a dishonest point of view, and they can't or won't do anything about it, so they weaken students' curiosity about sports. In addition, most people who teach physical education have shallow knowledge. They only know a few sporting events, and they may not be proficient in them. They lack in-depth knowledge and only their mechanical movements are seen by others every day. Therefore, many think that sportspersons are uneducated. Indeed, only focusing on the “body” of sports without also focusing on “education” or only paying attention to the form but not the substance will not do for a long time, and the fact of today's school sports is the same.

### Current situation of intelligent management of physical health of Chinese college students

At present, information technology closely links the world promoting the process of sports informatization and development of sports worldwide (14). The intelligent management of physical health has become an important way to improve the physical health level of college students and an important means to promote the intelligent services of college students' physical health. However, there are still many problems in the intelligent governance of college students' physical health, such as insufficient platform utilization, lack of resources, lagging research, and a low governance level.

#### Insufficient use of intelligent governance platforms for college students' physical health

According to our survey, although some Chinese universities have established an information platform for student physical health monitoring systems, the platform is only a decoration and has not been really used. Both the Ministry of Education and the General Administration of Sports have information technology centers, which are mainly responsible for monitoring and have been specifically set up to relay the macro-management and scientific decision-making efforts of ministries and commissions. Despite the establishment of sunshine sports and national fitness platforms, due to its outdated model lagging in products and services, their benefits are poor. How to reasonably combine school sports with informatization, build an intelligent governance platform for physical health in colleges and universities, and make full use of this platform to guide students' scientific sports and healthy life is an urgent problem that needs to be solved.

#### Lack of intelligent physical health service teaching resources for college students

For a long time, physical education teaching in colleges and universities has had a single structure, outdated content, traditional methods, and insufficient interactions. Teachers are the absolute owners of knowledge, and students can only learn from teachers in class. Therefore, the lack of communication and sharing in teaching makes it difficult to carry out educational and teaching activities creatively. The construction of physical health intelligent governance systems driven by big data has had great influence and significance. On the one hand, information-based physical education teaching resources can break through the traditional narrow teaching methods, letting the life experiences of teachers and students enter the physical education teaching process and rendering the classroom truly active. On the other hand, it can also change the status of learners in the physical education curriculum from passive cognitive audience members to knowledge-builders so as to transfer students' learning interests and play a positive role. At the same time, it can also broaden teachers' educational horizons, alter teachers' teaching concepts, and obtain the possibility of interaction and resource-sharing with various sports teaching resources, especially teaching material resources. The possibility of mutual cross-transformation of sports teaching resources inside and outside the school will also be increased, and an integrated sports teaching method inside and outside the curriculum will really be realized. Although many enterprises, colleges and universities, and individual teachers have carried out corresponding sports teaching resource construction projects by using big data, and networks, most have employed single sports material resource databases. At the same time, due to the various selection and use environments of sports materials, they sometimes have no way to start within their own schools or individual teachers in the selection process, and even need to be modified again; therefore, teachers must spend significant amounts of time and spiritual resources to do this kind of thing well. Sometimes, it goes against the original design and even impacts the improvement of school physical education teaching quality, which has no obvious effect on the improvement of college students' physical health. Colleges and universities should make full use of information technology, integrate physical education teaching resources, integrate network physical education courses, build high-quality and rich physical education teaching resources, and attain diversified physical education teaching processes and space. They must enable teachers to obtain physical education teaching resources they want in a limited time frame and encourage students to adopt exercise habits through an effective autonomous learning mode so as to finally achieve the purpose of improving their health (15).

#### The research on intelligent physical health services for college students lags behind

At present, there is still a lack of sports informatics specialty knowledge in China. The research on physical health and intelligent governance is mostly interdisciplinary research, while at the application level, it focuses on sports products on the market, mainly forming a new ecological environment of the Internet industry in the form of a platform, including mobile terminal applications such as sports fitness and running programs. Because China's sports intelligent governance started late and lacks relevant disciplines, there are those who understand sports but do not understand informatization, and those who understand informatization do not understand sports. Moreover, these mobile terminal products and information technology research fields are not well-integrated, and the technical content of products is generally not high. They mainly make profits through content and capital movement. Based on the Internet of Things, artificial intelligence competition training, physical education, physical health monitoring, and other research fields, there is still a large space to fill at present.

#### The level of intelligent governance of college students' physical health is low

According to the survey results, college students' physical health management services still lack the support of big data, mainly in three aspects. First, the college sports intelligent governance system is not enough. Only the venues in China's college sports intelligent governance at present have the “Internet of Things,” while construction at the infrastructure level remains focused on local support of facilities for activity service processes. To realize big data-driven college sports intelligent governance in China, we must establish a systematic and overall intelligent governance system based on intelligent technology and infrastructure. In addition, Chinese universities pay more attention to static information, while real-time and dynamic stream data information has not been widely used, and this data information is often necessary to support the school's intelligent movement behaviors. Second, the current results of big data analysis of college students' physical health have not been systematically organized and managed. In its current form, the existence of large quantities of college students' physical health information data increases the difficulty of obtaining big data analysis information from relevant institutions, which seriously interferes with the reasonable screening, identification, and mining of information data. Third, the current college students' physical health service system structure has been unable to meet the requirements of big data-driven intelligent development. At present, the collection, management, storage, and big data analysis of college students' physical health information and the school decision-making system have been unable to support decision-making through big data technology and cannot realize intelligent school physical education and intelligent physical health services.

### Construction of big data-driven intelligent governance system for college students' physical health

From the perspective of being big data-driven, this paper explores the development of a college students' physical health intelligent governance system. According to information ecological chain theory and guided by college students' physical health, a big data-driven college students' physical health intelligent governance system has been formed that can integrate infrastructure, health data resources, and business applications with students; explore the value of data intelligence in college students' physical health data resources by using big data analysis technology; provide learners with efficient, intelligent, accurate, and humanized intelligent sports consulting services; and realize integrated physical education inside and outside the classroom. Intelligent wearable devices are used to achieve physical fitness testing, sports monitoring, disease and health management, and dynamic adjustment of physical education teaching content. In combination with the physical health management system, a home-school linkage mechanism is established to master students' physical sports development, and psychological conditions with the use of emerging information technology. Personalized teaching and exercise regimens are pushed to truly achieve individualized teaching and accurate guidance and improvement of college students' physical health.

**Figure 1:**
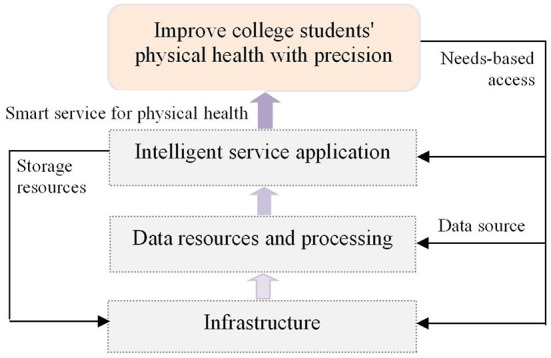
Data-driven intelligent governance system architecture of college students' physical health.

The data-driven intelligent governance system for college students' physical health includes three levels: school infrastructure, data resources and processing technology, and intelligent service application (see Figure 1). Among these, school infrastructure refers to the material foundation and technical support. The material foundation includes sensing, storage, computing, networks, and other equipment, and the technical support includes cloud computing, mobile Internet, the Internet of Things, artificial intelligence, and deep learning. Data resources are those available through big data technology and intelligent wearable devices about students' physical development, physical health, and movements at sports venues data mining. Data resources include physical health management data and student behavior data such as wisdom and are made available by machine learning, artificial neural networks, data visualization, and social network analysis means and methods for data processing to provide managers with intelligence data services. Smart administrators provide efficient, intelligent, accurate, and personalized smart sports services to college students through data-driven procurement (exercise information, good exercise methods), venue space-time optimization (field exercise experience), health knowledge discovery (knowledge transfer), and sports prescription alerts (16). This system can not only accurately guide and improve the physical health level of college students but also bring about the integration of teaching in and out of class.

#### Infrastructure: The supporting environment for the intelligent governance system of college students' physical health

**Figure 2:**
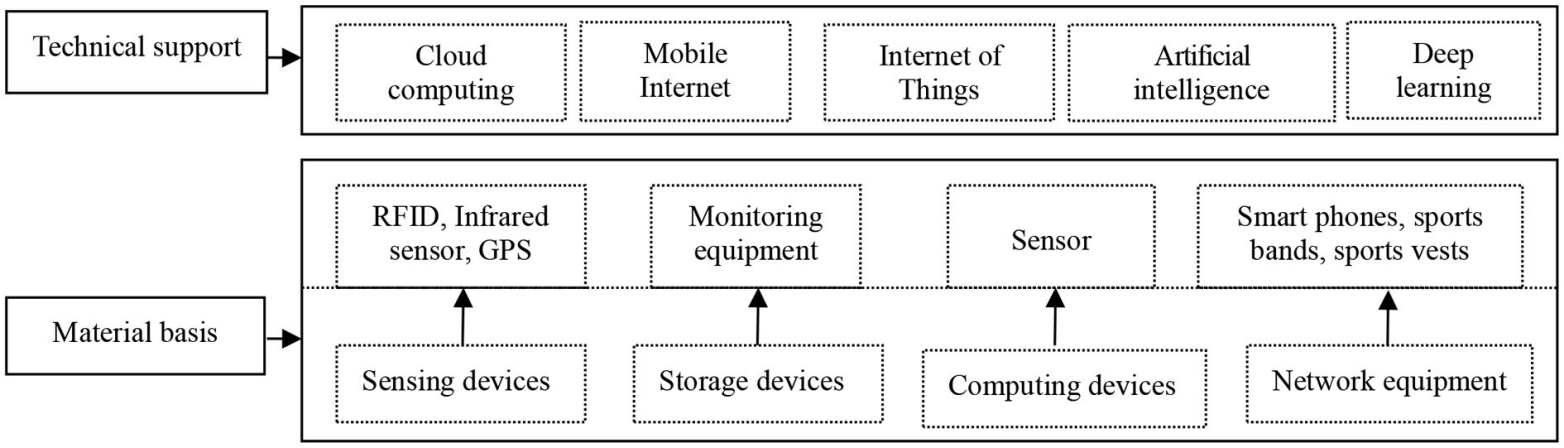
Infrastructure architecture.

The collection, storage, processing, organization, analysis, and application of big data depend on the support of a stable infrastructure framework. The infrastructure provides a guaranteed platform for college students' physical health and intelligent life for big data-driven research. Its main structure is composed of hardware equipment and information technology (see Figure 2). College students' physical health intelligent service facilities cover radiofrequency identification technology, monitors, sensors, smartphones or sports bracelets, and other sensing devices as well as various information technology facilities, including memory, computing equipment, network equipment, and so on. The complete layout of a big data analysis sensor identification system has the functions of in-depth understanding, detection, and capture of sports information data, operation status of stadiums and gymnasiums, and students' needs. It is a physique and fitness big data analysis system that can comprehensively perceive, intelligently screen, and upload in real-time. At the same time, hardware equipment with high performance, high bandwidth, and a large cache is an important material premise for the analysis, storage, analysis, and application of college students' physical health big data. It should offer a solid hardware foundation for solving new challenges, such as the diversification of data sensing ports, the scale of big data analysis resource centers, and the distribution of college students' physical health intelligent services. To realize big data analysis-driven college students' physical health and intelligent services, we must also combine new technologies, such as cloud computing technology, mobile Internet, the Internet of Things, artificial intelligence, and deep learning, which is also the key basis of big data analysis of Chinese college students' physical health. We can complete big data statistical analysis, visual data analysis, semantic analysis, and predictive data analysis and find the implied knowledge relationship and other basic functions in a large number of data analyses.

#### Data resources and processing technology: Data mining for intelligent governance system of college students' physical health

**Figure 3:**
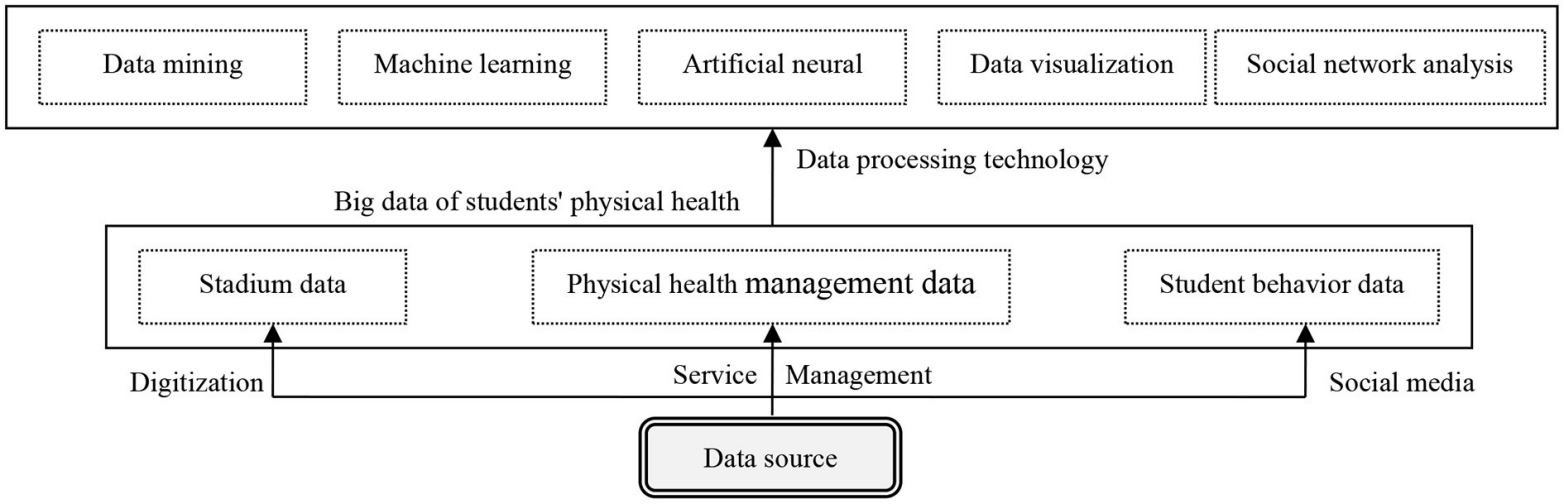
Data resources and processing architecture.

The data resources and management stage is responsible for the organization, management, and data analysis and mining of college students' physical health big data analysis, which is mainly composed of college students' physical health big data resources and big data processing technology (see Figure 3). College students' health big data is a mass of data formed during the process of students' daily physical exercise life and health management services. Generally, according to different data-formation methods, it is roughly divided into the following three types. The first is stadium database data, including venue reservation, sports knowledge base, online learning data, etc. The second is management and service data analysis. The data analysis generated during college students' daily sports includes resource construction status information records and radiofrequency identification system data analysis, access control information, monitoring information, and other Internet of Things end-user data analyses. In addition, it also involves business data analyses such as management consultation information records, training and guidance service information records, service quality evaluation feedback, and so on. The third is students' sports behavior data—that is, the behavior data left by students on the social platform, including evaluations, text, photos, videos, and so on. However, the volume of college students' physical health data is so huge, massive, and heterogeneous that new computing algorithms and protocols, more computing power, and new strategies are needed to properly and effectively combine and integrate data (17). The systematic construction of intelligent service capability is also inseparable from the support of relevant science and technology. The big data analysis of college students' physical health includes the data analysis of complex categories, such as text, pictures, sound, and videos, which requires the processing and exploration of international leading data mining technology. For example, text mining, natural language processing technology, and cognitive graphics are used to analyze text data; graphics recognition, speech recognition, video data analysis, and other means are used to analyze text, sound, and video big data; and data mining, social Internet, in-depth learning data analysis, and other means are used to analyze social media data (18, 19). To sum up, college students' physical health problems should be effectively managed and controlled through the flexible use of big data analysis, visual research, information mining, machine learning, artificial neural network, social Internet, and other analysis technologies so as to achieve the goal of maximizing the value of college students' physical health big data.

#### Intelligent service application: Value-shaping of intelligent governance system of college students' physical health

**Figure 4:**
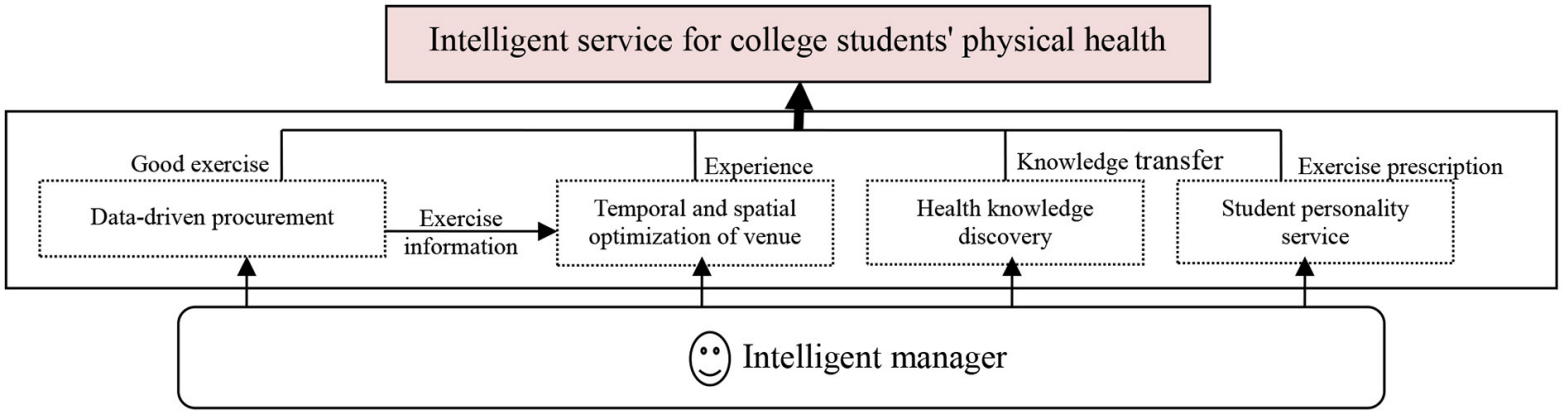
Service application architecture.

The intelligent service application stage is the top-level interactive port of the whole system, which directly interfaces with students and is composed of three elements: intelligent managers, students, and intelligent governance (see Figure 4). First, university intelligent managers use big data analysis methods, such as machine learning, data mining, image visualization, and artificial intelligence technology, to discover and process knowledge of college students' physical health big data and to impart professional knowledge to students. Second, college students will use the physical health intelligent management system to carry out higher-level physical exercise activities, and the formed data information will continue to enrich data resources to promote the vigorous development of college students' physical health management. In the ecological chain of intelligent governance of college students' physical health, intelligent administrators shoulder the mission of information decomposers and information disseminators, while students hold roles as both consumers and producers of information. They cooperate and coordinate with each other to jointly construct an important human resource element that is indispensable in the intelligent governance of college students' physical health. In addition, intelligent managers also mainly provide students with intelligent management of physical health of four types, including big data-driven procurement, venue space optimization, fitness knowledge discovery, and personalized services. Data-driven procurement services provide direct data resources for venue space optimization and health knowledge discovery. Health knowledge discovery and personalized services focus on the mining of physical health data and student sports behavior activity data, respectively, combining the in-depth exploration of professional knowledge and skills with the customized recommendation of professional knowledge and skills and continuing to promote the innovation of governance models, such as more accurate college students' physical health services and reference consultations, under the environment of big data and the Internet.

### Big data-driven intelligent governance strategy for college students' physical health

With the comprehensive development of physical health intelligent big data services, the wide application of the Internet of Things and artificial intelligence technology, the collaborative integration of multi-source heterogeneous information, and the gradual formation of intelligent sports atmosphere, a new service strategy has been put forward for the development of physical health intelligent service systems driven by big data. This paper applies the new generation of information technology, such as big data, the Internet of Things, and intelligent equipment, to improve the physical health of college students and suggests governance strategies, such as building an intelligent governance ecosystem of school sports, establishing a support system of school stadiums and gymnasiums, and building an integrated intelligent governance model of physical health.

#### Top-level design: Build an intelligent governance ecosystem of school physical education

The main application purpose of intelligent technology is to help establish a physical health intelligent environment that can mobilize people's creativity by coordinating the interaction between science and technology, resources, and people. While stimulating the physical health intelligent service ability driven by students' needs, it can attract potential students and promote the intelligent development and innovation of school physical education. A physical health intelligent management system can integrate students' physical health status into the construction of the campus sports network, resulting in a complex and integrated health ecosystem. In addition, the physical health intelligent service integrates big data analysis in the fields of physical education teaching, sports training, sports skills, after-school exercise, sports activities, academic research, and fitness management It integrates and links various sports environments, forms a network platform supporting health data analysis and decision-making, and helps the development and innovation of school sports. It also promotes the innovation of the campus sports network, and through this attracts more students to participate in the ranks of sharing sports and sports innovation. It encourages users to activate intelligent sports activities and intelligent sports services in a wider region and makes the physical health intelligent governance system truly become a network platform connecting cross-domain research, development, innovation, and various sports knowledge-intensive applications to obtain sustainable growth of new intelligent data in the campus sports network.

#### Interconnection of all things: Constructing the supporting system of school stadiums and gymnasiums with intelligent perception

The Internet of Things is a network of communication between people and objects and between objects, which is a perception technology for intelligent stadiums and gymnasiums. By applying high and new technologies like big data technology, Internet of Things technology, and artificial intelligence to the design and construction of campus stadiums and gymnasiums, the services of campus stadiums and gymnasiums will become more automatic and intelligent. By taking advantage of the advanced technology and equipment advantages of intelligent sensing systems and cognitive equipment, virtual reality, and intelligent robots in the Internet of Things, we can further enhance personalized, convenient, and efficient physical health services so as to make campus stadiums truly automated. For example, in terms of intelligent sensing systems, campus stadiums and gymnasiums can promote the convenience of venue resources and space management through the effective use of Internet of Things sensors. First, the use of a radiofrequency identification system, sports software, indoor navigation robots, and other advanced technical means can facilitate site reservation and management so that students can easily access site materials. Second, connections and communication among people, machines, and mobile networks can be realized through machine-to-machine technology. The module that can monitor the operation parameters of a distribution network can be installed in the power equipment of stadiums and gymnasiums to realize the real-time monitoring, control, management, and maintenance of distribution systems. Third, face recognition, infrared sensors, cameras, and other technical means help to understand students' sports behavior; infer students' needs through students' sports behavior data; and provide data support for students' personalized sports suggestions and services. In addition, through augmented reality, virtual reality, and other technical means, students can also have virtual and intelligent space service experiences of virtual and real integration (20).

#### Action practice: Build an integrated intelligent governance model of physical health

Through the Internet, mobile Internet, big data, artificial intelligence, and other new-generation information technologies and knowledge, we can carry out intelligent physical education and training, after-school physical exercise, physical health monitoring, and evaluation, and campus sports events. We can form an integrated physical health intelligent governance model that can accurately guide, manage, supervise, and improve the physical health of college students. Intelligent physical education is mainly embodied in information-based physical education teaching design, mobile education, and ubiquitous learning. Through big data, we can build physical education courses and online, offline, and hybrid physical education courses; independently design and develop school-based physical education curriculum resource databases, design learning methods, teaching evaluations, and other modules. It can help realize resource appreciation through the screening, evaluation, reorganization, and integration of curriculum experiences, which can meet students' online physical education learning at any time. Through big data technology, we can also carry out the integrated application of mobile teaching platforms, smart terminal teaching, and university sports services. It can be used to develop mobile teaching tools of wearable devices and university sports public service products based on big data analysis to carry out in-depth teaching research, technical innovation, system incubation, detection and evaluation, application demonstration, and other physical health intelligent services. In terms of students' intelligent after-school physical exercise and physical health monitoring and evaluation, we can use big data, the Internet, and other analysis technologies to comprehensively and dynamically manage students' physical and mental health, mastery of sports skills, and extracurricular physical exercise. The administrator transmits the sports load, physical condition, motion trajectory, and other data collected as front-end data to teachers and students through sensor devices such as smartphones, sports bracelets, and sports vests while simultaneous background calculations of the server and the global positioning system are ongoing. Teachers and systems set the amount of exercise and monitor the situation of extracurricular exercise according to the specific situation and measurement data, then formulate exercise schedules and provide accurate guidance to students so as to reduce sports injury, realize scientific exercise and learning, and accurately serve and manage students' physical health., The new generation of information technology may be used to build the school competition platform, and an information system integrating competition qualification examinations, registration data, competition arrangements, scoring systems, and score information. Competition data analysis can be established within the platform. With this approach, both teachers and students can view the competition results in real-time; the competition situation can be recorded, analyzed, and managed. This approach allows managers, teachers, and students to master students' sports level and physical health in real-time, and data support for dynamic adjustment of physical health intelligent governance strategies can be provided. The college and students can be urged to meet relevant indicators to form an effective intelligent and dynamic integrated physical health governance system.

## Conclusions

This study, based on the realistic background of big data, the Internet, mobile Internet, and artificial intelligence, sought an angle to explore the precise path and methods for improving the physical health of university students, which is a relatively new research field. The research method of traditional improvements to physical health and their innovation is of great significance to promote college students' physical health levels. The intelligent governance system of physical health driven by big data can quickly and comprehensively understand the dynamic state of the physical health of college students, while at the whole-time monitoring sports, disease health management, and dynamic adjustment of physical exercise content. Combined with a physical health management system to establish the linkage mechanism, the government, school, society, and family can use emerging information technology to master students' sports, physical development, and psychological situation; personalize push exercise prescriptions; realize students' aptitude, and precisely promote the physical health of college students to solve the problem of Chinese college students' declining physical health (21). For example, relying on cloud platforms such as the Lejian Sports app, the WeChat public platform, QQ, and online live broadcasts, the University of Electronic Science and Technology built an integrated interactive intelligent physical health management system that combines online plus offline and in-class plus after-class data. Club teaching or online teaching and practice requirements are mainly implemented for students in class, and offline guidance and cloud interactive practice are implemented for students after class. Analysis of the sports data platform revealed that, since the construction of intelligent governance systems for physical health, the daily number of extracurricular sports has increased greatly, the qualified rate of physical health has improved year by year, and the physical quality level has also improved significantly.

It is suggested that schools at all levels should make full use of modern information science and technology in the future, monitor the physical health of college students, monitor their physical activities, set up a physical activity guidance and service platform, guide students to exercise scientifically, and strive to improve the physical health level of students. How to make full use of big data, artificial intelligence, intelligent equipment, and the Internet of Things as part of a new generation of information technology to improve students' physique health as well as to create intelligent physical health service models for precise improvements in students physical health patterns remain future research directions to be explored.

## Data availability statement

The original contributions presented in the study are included in the article/supplementary material, further inquiries can be directed to the corresponding author.

## Ethics statement

Ethical review and approval was not required for the study on human participants in accordance with the local legislation and institutional requirements. The patients/participants provided their written informed consent to participate in this study.

## Author contributions

CD and QY studied the current situation of college students' physical health and its informatization governance problems, the construction of governance system, and intelligent governance strategies. GL, ZZ, and YL participated in the exchange and discussion. CD drafted and revised the paper. All authors contributed to the article and approved the submitted version.

## Funding

This research was supported by the Basic Scientific Research Operating Expenses Project of the Central University of China (Grant No. ZYGX2019J143), the Educational Research Project of Sichuan Province (Grant No. SCJG20A169), the Ideological and Political Demonstration Course Construction Project of UESTC (Grant No. 2021KCSZ0196), and the Relay Action Plan for Young Teachers in Philosophy and Social Sciences of UESTC in 2021.

## Conflict of interest

The authors declare that the research was conducted in the absence of any commercial or financial relationships that could be construed as a potential conflict of interest.

## Publisher's note

All claims expressed in this article are solely those of the authors and do not necessarily represent those of their affiliated organizations, or those of the publisher, the editors and the reviewers. Any product that may be evaluated in this article, or claim that may be made by its manufacturer, is not guaranteed or endorsed by the publisher.
